# Novel Management of Post-laparoscopic Sacrocolpopexy-Associated Overactive Bladder: A Combined Approach of Vaginal Natural Orifice Transluminal Endoscopic Surgery (vNOTES) Mesh Removal and Fotona Laser Therapy

**DOI:** 10.7759/cureus.79277

**Published:** 2025-02-19

**Authors:** Nobuo Okui, Machiko Okui

**Affiliations:** 1 Urogynecology, Yokosuka Urogynecology and Urology Clinic, Yokosuka, JPN; 2 Dentistry, Kanagawa Dental University, Yokosuka, JPN

**Keywords:** fotona laser therapy, laparoscopic sacrocolpopexy, mesh complications, non-ablative erbium yag laser, overactive bladder, vaginal natural orifice transluminal endoscopic surgery

## Abstract

This case report presents a novel approach for managing overactive bladder (OAB) syndrome following laparoscopic sacrocolpopexy (LSC) using vaginal natural orifice transluminal endoscopic surgery (vNOTES) and Fotona laser therapy. A 73-year-old woman with severe OAB syndrome and pelvic pain after LSC underwent mesh removal via vNOTES. Despite the initial improvement in OAB symptoms, the patient continued to experience persistent urinary issues. Subsequent treatment with Fotona's non-ablative erbium:yttrium-aluminum-garnet (Er:YAG) laser therapy, including Vaginal Erbium Laser (VEL) and Urethral Erbium Laser (UEL), led to the complete resolution of OAB symptoms. The patient's Overactive Bladder Symptom Score (OABSS) significantly improved following combined vNOTES and laser therapy. Follow-up assessments revealed sustained improvements in bladder function and quality of life. This case highlights the potential of combining vNOTES for mesh removal and Fotona laser therapy for managing post-LSC complications, particularly in cases in which mesh-related issues contribute to persistent OAB syndrome. The successful outcome, as evidenced by symptom resolution and improved OABSS, suggests that this approach may offer a viable solution for patients experiencing persistent OAB syndrome following LSC, especially when conventional treatment fails. This report contributes to the limited body of evidence on managing LSC-related OAB syndrome and introduces a promising treatment protocol using Fotona laser therapy, which merits further investigation in larger studies focused on OAB management after pelvic floor surgery.

## Introduction

Laparoscopic sacrocolpopexy (LSC) with polypropylene mesh insertion has become the standard treatment for pelvic organ prolapse (POP) [1]. However, this procedure is associated with complications, including pain, overactive bladder (OAB), and other urogenital issues [2,3]. The removal of polypropylene mesh, which is often necessary to address these complications, presents significant challenges owing to the risks of postoperative complications and technical difficulties [4,5].

Long-term studies have shown that complications following LSC can persist for years, with reoperation rates for various issues, including mesh-related problems, reaching up to 5.1% over a four-year period [6]. These findings highlight the need for effective management strategies for post-LSC complications, particularly for OAB. Although traditional approaches, such as vaginal and open abdominal surgeries, have been employed for mesh removal, they are often hindered by limited visibility and accessibility at mesh fixation sites. To address these challenges, vaginal natural orifice transluminal endoscopic surgery (vNOTES) has emerged as a promising technique for mesh removal [7,8]. This minimally invasive approach offers enhanced visibility and reduced invasiveness compared with conventional methods. Despite the growing adoption of vNOTES, there is a paucity of research specifically addressing its application to LSC mesh removal and subsequent management of OAB. This gap in the literature underscores the need for more comprehensive studies on the efficacy and safety of vNOTES in this context [7].

Furthermore, the management of post-removal complications, particularly persistent OAB symptoms, remains a significant challenge. In this regard, the use of Fotona laser treatment presents a novel approach. Recent studies have shown promising results with the use of non-ablative erbium:yttrium-aluminum-garnet (Er:YAG) laser treatment for various urogenital conditions, including stress urinary incontinence and OAB [9-11].

This case report describes the successful use of vNOTES for LSC mesh removal in a patient with OAB. It also describes the subsequent use of Fotona laser treatment to address persistent OAB symptoms, offering new insights into the management of post-mesh removal complications. To our knowledge, this is the first report documenting the successful use of Fotona laser therapy to improve OAB symptoms following mesh removal. Our findings contribute to the growing body of evidence supporting the use of vNOTES in mesh removal surgeries and introduce an innovative approach for treating postsurgical OAB.

## Case presentation

A 73-year-old woman presented with severe OAB syndrome and pelvic pain after LSC. Her medical history included a hysterectomy for uterine prolapse seven years prior, followed by the development of cystocele three years later. She subsequently underwent LSC, after which she experienced persistent OAB symptoms and vaginal pain. The patient reported ongoing pelvic pain, with a preoperative Visual Analog Scale (VAS) score of 7/10, indicating severe discomfort. Following LSC, after which she developed chronic urogenital discomfort and underwent various treatments, including medication for OAB. However, these interventions were ineffective in managing her symptoms. Notably, hormone replacement therapy, which is sometimes used to address urogenital symptoms in postmenopausal women, was contraindicated in this patient owing to a history of thrombosis. This limitation further complicated her treatment options. Despite treatment with OAB medication, her symptoms remained poorly controlled, significantly impacting her quality of life. The patient's Overactive Bladder Symptom Score (OABSS) remained consistently high, indicating the severity and persistence of her condition. After a thorough discussion and consideration of her limited options, the patient understood that mesh removal would be the most effective treatment for her ongoing symptoms. Given the complexity of the patient and the need for a minimally invasive approach, the patient was referred for mesh removal via vNOTES. This technique was chosen for its potential to offer improved visibility and access to the mesh, while minimizing surgical trauma and the risk of further complications.

Laboratory investigations demonstrated normal hematological and biochemical parameters. The complete blood count showed white blood cells at 5050/μL, hemoglobin at 13.3 g/dL, and platelet count at 22.8 x10^4^/μL. Coagulation studies were unremarkable with a prothrombin time-international normalized ratio (PT-INR) of 1.01 and an activated partial thromboplastin time (APTT) of 37.2 sec. Liver function tests were within normal ranges, with an aspartate aminotransferase (AST) at 19 U/L and an alanine aminotransferase (ALT) at 14 U/L. Kidney function was also normal, with creatinine at 0.49 mg/dL. The patient's glycemic control was good, as indicated by an HbA1c of 5.2%. The cardiac evaluation revealed mild QT prolongation on ECG, while the chest X-ray showed a cardiothoracic ratio of 48.4% with clear lung fields.

At our clinic, we conducted assessments using OABSS, Overactive Bladder Questionnaire (OAB-q), vaginal bacterial culture, urodynamic study, pressure-flow study, MRI, and cystoscopy. We determined that the mesh inserted during LSC caused a severe OAB and proceeded with mesh removal via vNOTES.

**Figure 1:**
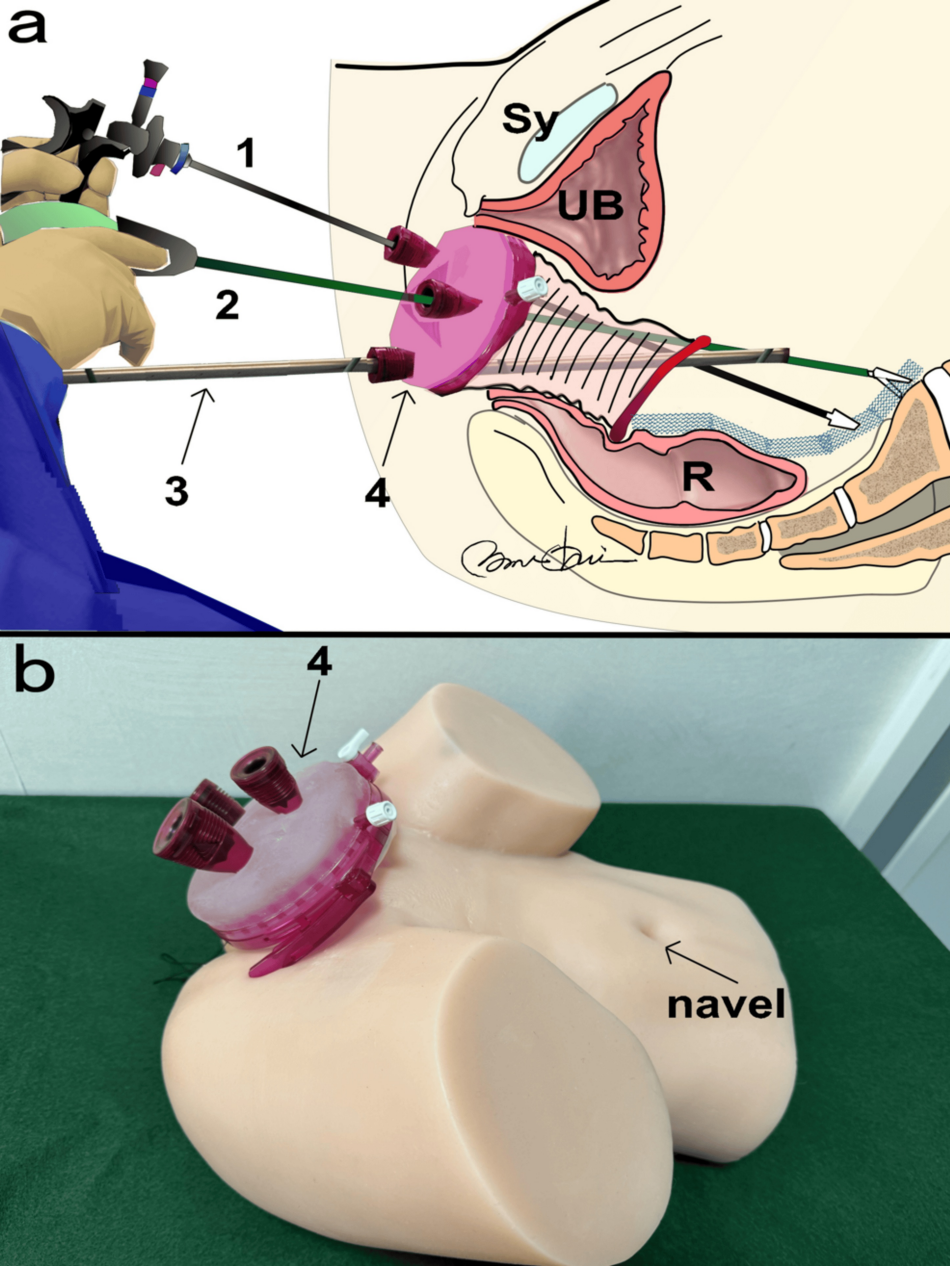
The Vaginal Natural Orifice Transluminal Endoscopic Surgery (vNOTES) UB: Bladder; Sy: Pubis; R: Rectum a: Mesh removal illustrated; b: GelPoint™ Access Platform using a silicone model 1: Ultrasonic coagulation cutting device SONICBEAT; 2: HICURA-type grasping forceps; 3: Endoscope by OLYMPUS; 4: GelPoint™ Access Platform Illustration by Nobuo Okui

Figure 1 shows the vNOTES system. The Access Platform GelPoint™ device (Applied Medical, Rancho Santa Margarita, California), endoscopic carbon dioxide insufflation device OLYMPUS UCR (Olympus Corporation, Tokyo, Japan), and HICURA-type grasping forceps (Olympus Corporation) were used in the procedure. A GelPoint™ device was inserted, and a CO_2_ gas pressure of 8 mmHg was used. A standard 10 mm, 0° rigid laparoscope was inserted through the camera trocar at the 6 o'clock position, with standard laparoscopic instruments inserted through the other two trocars. The laparoscopy equipment included the VISERA ELITE II high-intensity light source unit (OLYMPUS CLV-S190), endoscopic carbon dioxide insufflation device (OLYMPUS UCR), and HICURA-type grasping forceps. The patient was administered general anesthesia and placed in a supine position. Antiseptic drapes were applied to the perineal and abdominal areas to prepare for a potential switch to laparotomy or laparoscopy. A urinary catheter was inserted at the beginning of the procedure, and 2 g of cefazolin was administered intravenously as antibiotic prophylaxis. The first stage of the surgery involved following a standard vaginal mesh removal procedure, which included making a midline incision in the central part of the vaginal anterior wall to selectively separate the mesh from the tissue while preserving blood vessels and fascia. The mesh arms that were inserted into the closed cavity through the vaginal anterior wall were removed, guided by the thinning of the vaginal wall along the arms. The partial detachment of the mesh arms facilitated easy removal. The second stage involved making a 360-degree incision at the vaginal blind end (formerly the site of the uterus), followed by arterial hemostasis.

**Figure 2:**
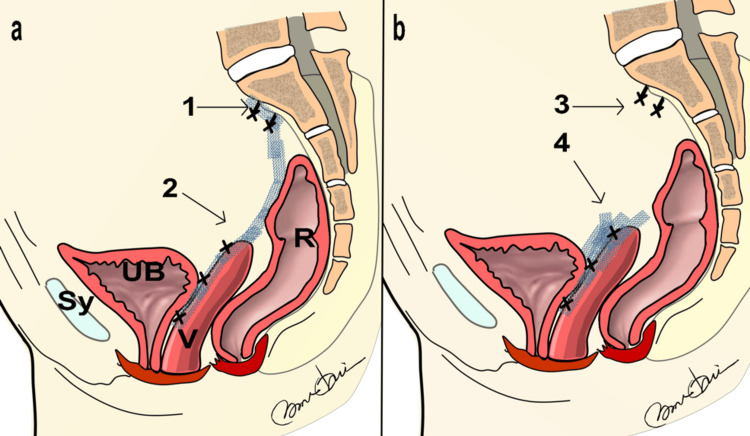
Planned Mesh Placement Location vs. Actual Mesh Location a: Illustration of polypropylene mesh inserted as planned; b: Illustration of polypropylene mesh where the sacral fixation has come off and formed adhesions in an unintended area 1: Sacral fixation area; 2: Vaginal stump fixation area; 3: Sacral fixation area (dislodged); 4: Area where polypropylene mesh overlaps and forms clumps UB: Bladder; Sy: Pubis Illustration by Nobuo Okui

The images demonstrate the mesh removal process using the minimally invasive vNOTES technique. As shown in Figure 2, the anatomy clearly depicts the site where the mesh was inserted during LSC. During surgery, the mesh was placed in the vesicovaginal space and secured to the bladder using absorbable sutures, with the end of the mesh attached to the sacrum. However, during mesh removal through vNOTES, the mesh overlapped in the vesicovaginal space, and the overlapping mesh at the site of the previous hysterectomy (the vaginal stump) exhibited inadequate granulation tissue formation.

**Figure 3:**
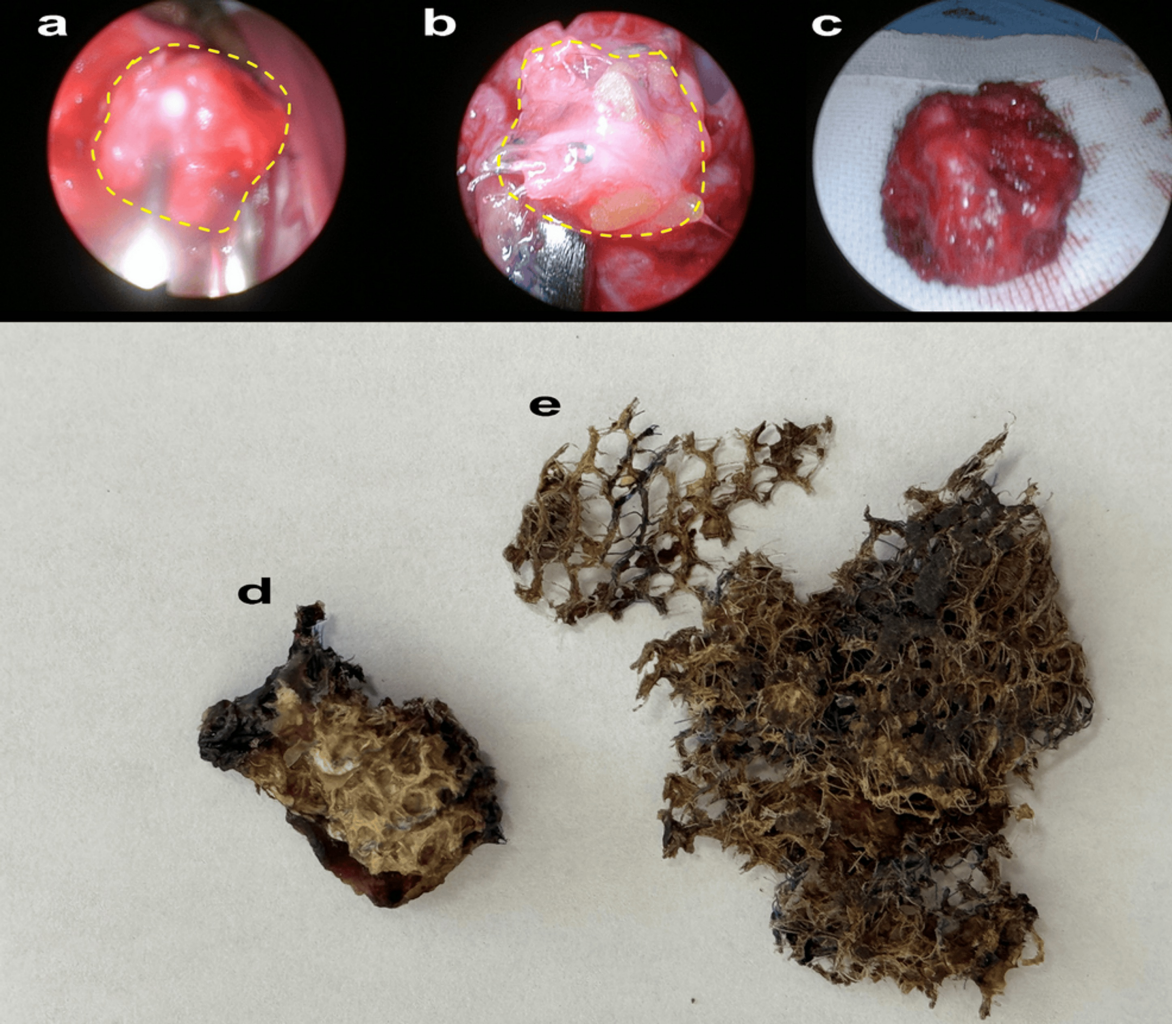
Observations and Specimens From vNOTES Mesh Removal a: Poor granulation tissue exhibiting elasticity and hardness, identified between the bladder and the vaginal stump (highlighted in yellow). b: Tissue excised from the area shown in (a) after making a central incision. c: The excised tissue specimen corresponding to (b), prior to further examination. d: A detailed, macroscopic view of the excised tissue shown in (c), highlighting its texture and structure. e: Additional mesh fragments that were extracted during the procedure, showcasing the extent of mesh removal. vNOTES: vaginal natural orifice transluminal endoscopic surgery

Figure 3 shows the findings of the vNOTES procedure, highlighting the challenges encountered during mesh removal. The area surrounding the mesh is notably fragile and prone to significant bleeding, with the mesh exerting direct pressure on the bladder. No large abscesses were observed during the procedure. However, the area around the mesh was characterized by strong adhesions, requiring careful and meticulous dissection to avoid damaging the surrounding nerves. During the dissection, substantial bleeding was encountered, making it necessary to perform repeated irrigation with saline solution to maintain visibility and a clear surgical field. In Figure 3a, elastic, hard, poor granulation tissue is observed in the area connecting the bladder and the vaginal stump, as indicated within the yellow dashed line. Figure 3b shows the poor granulation tissue dissected after an incision was made at the center of Figure 3a. Figure 3c presents the resected specimen from Figure 3b. Figure 3d provides a macroscopic view of the specimen shown in Figure 3c, and Figure 3e shows the additional parts of the mesh that were removed.

**Figure 4:**
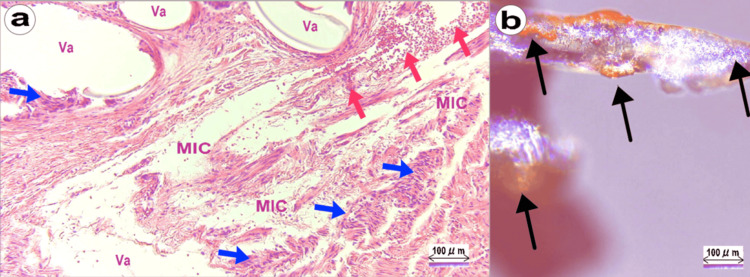
Histopathological Analysis of Resected Tissue and Mesh Fragment a: Histological section showing vacuoles and tissue damage associated with the mesh (H&E stain, 40x magnification, captured using the Olympus System Microscope BX43 (Olympus Corporation, Tokyo, Japan)). b: Close-up view of the removed mesh under a stereo microscope, highlighting frayed fibers (same magnification as (a), observed using the Olympus SZ61 stereo microscope (Olympus Corporation)). Va: Vacuoles formed by polypropylene mesh fibers; MIC: Mesh-induced contusion; Blue arrows: Aggregates of foreign body giant cells; Red arrows: Hemorrhage

Figure 4 illustrates the pathological examination of the resected specimen along with images of the mesh. Figure 4a shows tissue sections stained with hematoxylin and eosin (H&E), captured at an optical magnification of 40x using an Olympus System Microscope BX43 with the objective lens LPLN10X (Olympus Corporation). During the staining process, the polypropylene material of the mesh dissolved, leaving behind numerous vacuoles (Va) that corresponded to the spaces where the mesh fibers were originally located. Additionally, the image reveals areas where the tissue has been crushed due to the mesh (MIC), abscesses (indicated by blue arrows), and hemorrhage (indicated by red arrows). A scale bar of 100 μm is provided for reference, and Figure 4b shows the removed mesh observed under a stereo microscope SZ61 set (Olympus Corporation) at the same magnification as Figure 4a for comparative purposes. The mesh fibers appeared partially frayed, with inflammatory tissue adhering to these damaged regions (indicated by black arrows). Of particular note is the observation that, while the mesh fibers are approximately 200 μm in diameter as seen in Figure 4b, the vacuoles left by the mesh in Figure 4a vary in size, indicating a range of tissue responses to the mesh material.

**Figure 5:**
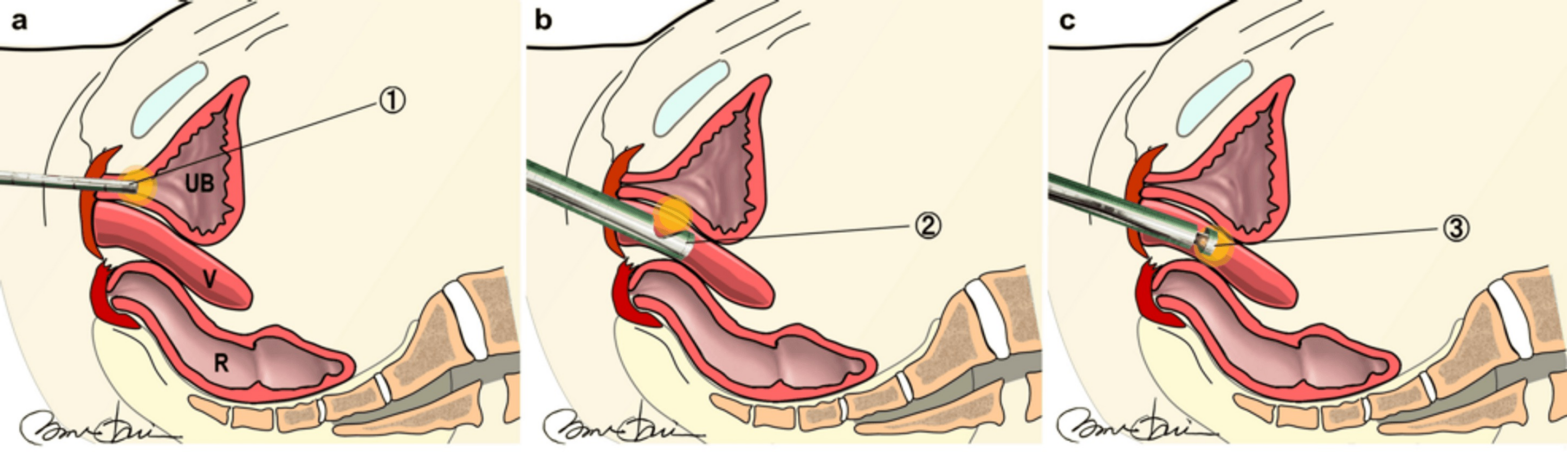
Fotona Laser Treatment a: UEL step (whole urethral laser irradiation with R09-2Gu) b: VEL step (laser irradiation of anterior vaginal wall by PS03) c: VEL step (whole vaginal laser irradiation by R11) 1: R09-2Gu intraurethral adapter 2: PS03 90° angular golden mirror titanium adapter 3: R11 360° circular golden mirror titanium adapter UB: Bladder; V: Vagina; R: Rectum; UEL: Urethral Erbium Laser; VEL: Vaginal Erbium Laser Illustration by Nobuo Okui

Figure 5 shows the Fotona laser treatment that was performed on the patient. This procedure was necessitated by the persistence of OAB symptoms, which continued even three months after the vNOTES surgery. The decision to use Fotona laser treatment was made in response to these ongoing urinary issues, indicating that the initial surgery had not fully resolved the patient's bladder dysfunction. The Fotona laser treatment incorporates several procedures, including Urethral Erbium Laser (UEL) and Vaginal Erbium Laser (VEL) treatments. For the UEL, after residual urine was removed from the bladder using a catheter, an R09-2 Gu laser probe, specifically designed for the urethra, was employed. The handpiece was connected to an Erbium YAG laser (SP Dynamis Fotona d.o.o, Ljubljana, Slovenia). The laser settings were configured to R09-2 Gu, SMOOTH mode, 1.4 Hz, 1.5 J/cm², and four stacks from the urethral meatus to the proximal end in 2.5 mm increments (Figure 5a). This procedure was repeated four times. For VEL, laser irradiation was performed using the same laser, starting with VEL and then proceeding to UEL. The equipment used included a specialized glass vaginal speculum made for laser probes and handpieces (PS03 and R11, respectively). Each handpiece was connected to an SP Dynamis laser. During the VEL step, a glass speculum was inserted into the vagina, and the anterior vaginal wall was scanned with a PS03 laser probe using a 7 mm spot size, pulse fluence of 6 J/cm², and a frequency of 2.0 Hz (Figure 5b). The area was irradiated at 5 mm intervals, and this procedure was repeated three times. Following this, the R11 laser probe was used to apply laser treatment at 5 mm intervals along the entire 360-degree circumference of the vaginal canal. This procedure involved a 7 mm spot size, pulse fluence of 3.00 J/cm², and a frequency of 2.0 Hz (Figure 5c). This process was repeated twice. After the procedure, patients were advised to maintain proper hygiene in the treated area.

Next, the progress of various tests for this patient will be explained, comparing the results at three stages: pre-vNOTES, post-vNOTES (three months after vNOTES), and post-Fotona laser treatment (three months after the Fotona laser).

MRI

**Figure 6:**
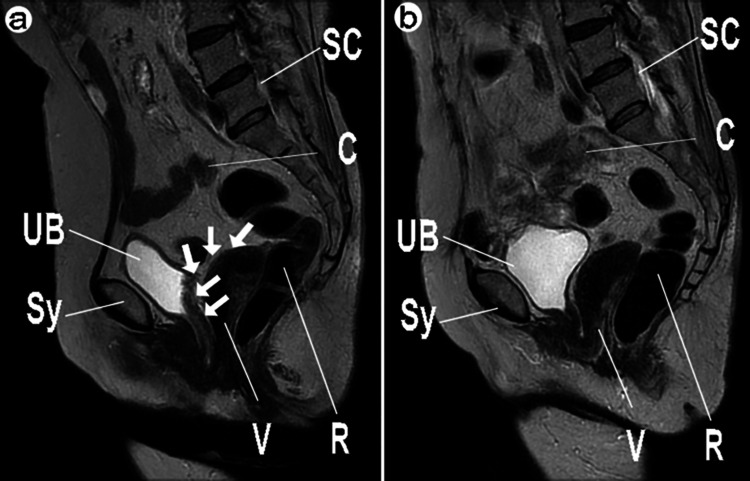
Pre- and Post-vNOTES MRI Imaging Showing Mesh Localization and Removal a: Before vNOTES procedure b: After mesh removal via vNOTES 1.5T MRI T2-weighted sagittal view, repetition time (TR) 4500 ms, flip angle 140 degrees, echo time (TE) 100 ms, slice thickness 3 mm, interval 3.5 mm, field of view (FOV) 32 x 36 cm, matrix 320 x 320 (Signa Creator, GE Healthcare, Chicago, USA). Arrows: Localization of the polypropylene mesh, notably clumped at the vaginal stump. UB: Urinary bladder; Sy: Symphysis pubis; SC: Spinal canal; C: Small intestine; R: Rectum; V: Vagina; vNOTES: vaginal natural orifice transluminal endoscopic surgery

Figure 6a shows that the MRI indicates the localization of the polypropylene mesh inserted during LSC. Additionally, an MRI in the horizontal section revealed that the urethra's vertical diameter was normal at 22 mm, although the anteroposterior diameter was unclear owing to curvature. The distance from the pubic symphysis to the coccyx was 83 mm and the distance to the anterior rectal wall was 32 mm, both within normal limits. However, vascular dilation was observed in the surrounding area, and the pelvic inlet diameter was slightly enlarged to 123 mm. These MRI findings, along with mild bladder wall thickening and high signal intensity on diffusion-weighted imaging, suggested a possible cystocele. Additionally, a discontinuity in the levator ani muscle was noted on the right side, but no ascites were present, and the spinal canal diameter was maintained. Figure 6b shows the MRI taken three months after the mesh removal via vNOTES.The cystocele and prolapse of the surrounding fat layers have improved, and there are no abnormalities in the urethra or levator ani muscle. There are no ascites.

Urodynamic study and pressure flow study

**Figure 7:**
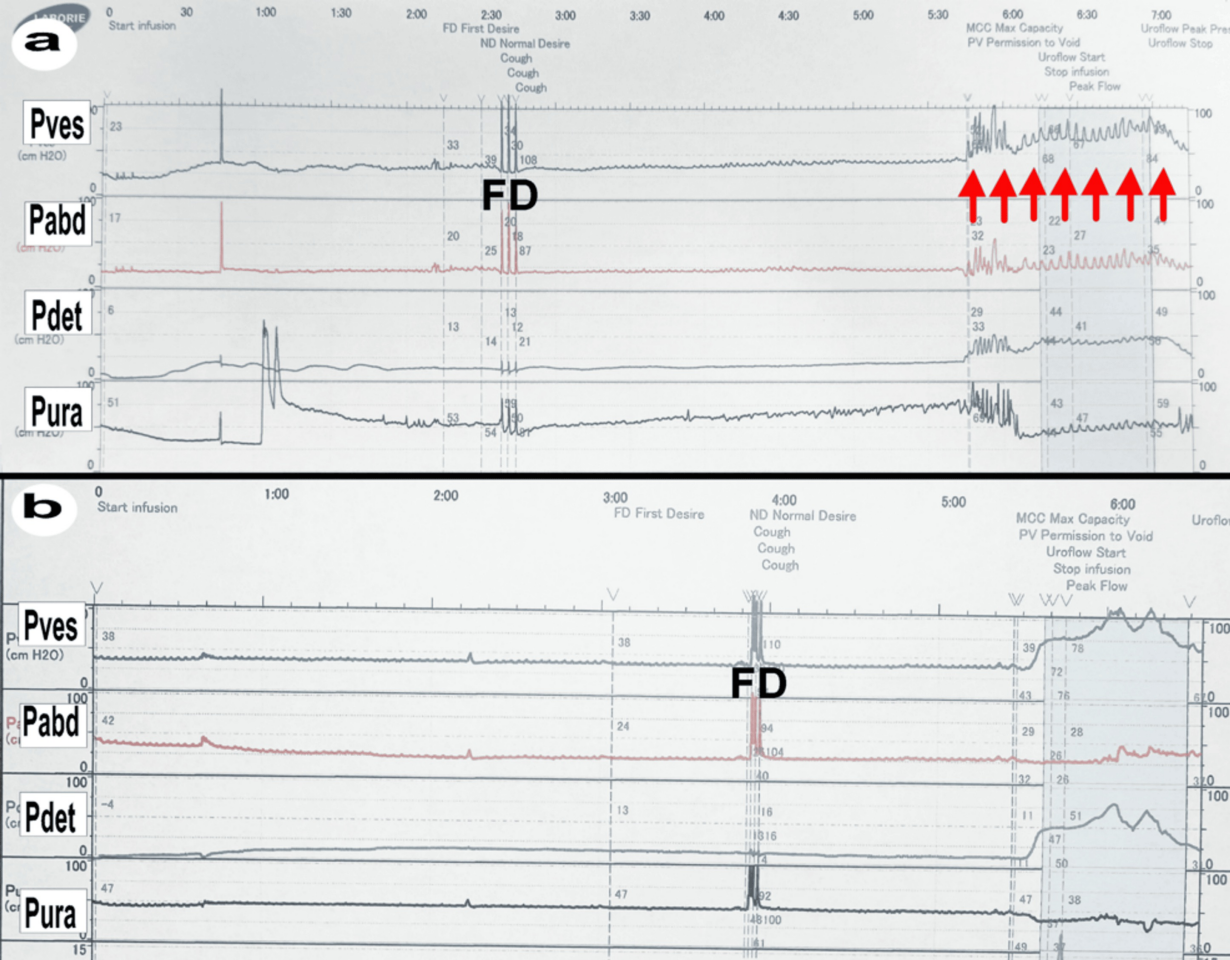
Urodynamic Study Results Before vNOTES a: Pre-vNOTES; b: Post-Fotona laser Y-Axis Parameters and Units: Pves: Intravesical pressure (cmH_2_O); Pabd: Abdominal pressure (cmH_2_O); Pdet: Detrusor pressure (cmH_2_O); Pura: Urethral pressure (cmH_2_O) X-Axis Parameter and Unit: Time (minutes) Printing Format: The format of the printout adheres to the guidelines of the Japanese Urological Association for urodynamic testing notation. Displayed Terms on Top of the Graph (Each term is printed at the point when the event occurs): Start infusion: Indicates the beginning of bladder filling. FD (First Desire): The point at which the patient first feels the desire to void. ND (Normal Desire): The point where a normal urge to void is felt. MCC (Max Capacity): Maximum bladder capacity. PV (Permission to Void): Point at which the patient is allowed to void. Uroflow Start/Stop: Start and stop of urine flow measurement. Peak Flow: Maximum urine flow rate. Red arrows mark the presence of involuntary contractions. The other parameters displayed on the screen are reference values and are therefore omitted from this discussion. vNOTES: vaginal natural orifice transluminal endoscopic surgery

Figure 7a shows the urodynamic study conducted before the vNOTES procedure. This study demonstrates a delay between the first urge to void and the actual voiding event. When the bladder is fully filled with saline, involuntary bladder contractions occur, corresponding to irritation and pain associated with the mesh inserted during LSC. In terms of pressure, intravesical pressure (Pves), abdominal pressure (Pabd), and detrusor pressure (Pdet) indicate that normal voiding urges do not elicit significant responses. However, the widespread mesh at the bladder-vaginal junction seems to stimulate the surrounding organs, causing discomfort. The Pura (urethral pressure) shows that strong involuntary bladder contractions generate signals to the urethra, which is consistent with clinical observations of both bladder and urethral pain during urination. The maximum flow rate recorded was 5.2 mL/sec, which was lower than expected, suggesting either a possible obstruction or weak detrusor muscle activity. The average flow rate was 3.4 mL/sec, with a voiding time of 44.6 seconds, indicating potential voiding dysfunction. The flow time was 43.3 seconds, and the post-void residual (PVR) volume was 164.0 mL, indicating incomplete bladder emptying. The peak voiding pressure is 49.5 cmH_2_O, with an average pressure of 44.6 cmH_2_O, an opening pressure of 41.6 cmH_2_O, and a closing pressure of 49.0 cmH_2_O. The maximum cystometric capacity (MCC) before treatment was 240 mL. These findings suggest a high-pressure voiding pattern, likely due to detrusor overactivity caused by the mesh. After vNOTES, the patient's bladder function showed significant improvement. The PVR volume decreased from 164.0 mL preoperatively to 32.0 mL post-vNOTES, indicating improved bladder emptying efficiency. Figure 7b shows the urodynamic study after Fotona laser treatment, highlighting the significant improvements in bladder function and control. The data reveal that the bladder no longer exhibited involuntary contractions when filled with urine, and the first urge to void improved to 152.5 mL, indicating better bladder control and increased capacity. MCC increased to 271.4 mL, reflecting enhanced bladder storage function. The voiding efficiency also improved, as indicated by a peak flow rate of 19.7 mL/sec and an average flow rate of 9.3 mL/sec, suggesting effective bladder emptying post-treatment. Additionally, the peak detrusor pressure during voiding was 49.7 cmH_2_O, showing an adequate but not excessively high-pressure voiding pattern, which points to improved bladder function without signs of detrusor overactivity. At this stage, the PVR volume further decreased to 12.0 mL, indicating near-complete resolution of incomplete bladder emptying. This reduction in PVR may have contributed to the resolution of OAB symptoms by minimizing residual urine-induced bladder irritation. These findings collectively demonstrate that vNOTES and Fotona laser therapy effectively reduced symptoms, such as urgency and pain, providing significant improvements in both bladder stability and overall urinary function.

Evaluation for pain and OAB

The patient's pelvic pain was assessed using the VAS. Preoperatively, the patient reported a VAS score of 7/10, indicating severe discomfort. At three months post-vNOTES, the pain score decreased to 3/10, reflecting moderate improvement. Following Fotona laser treatment, the pain further improved to 1/10, suggesting near-complete resolution of discomfort. Table 1 shows a comparative analysis of OAB symptoms and their impact on quality of life across pre-vNOTES, post-vNOTES, and post-Fotona laser treatments.

**Table 1:** Overactive Bladder Symptom Severity and Quality of Life Scores Pre- and Post-vNOTES OABSS: Overactive Bladder Symptom Score; OAB-q SS: Overactive Bladder Questionnaire Symptom Score; OAB-q HRQL: Overactive Bladder Questionnaire Health-Related Quality of Life; vNOTES: vaginal natural orifice transluminal endoscopic surgery The total score ranges and severity classifications for each scale are as follows: OABSS (Overactive Bladder Symptom Score): Range 0-15. - Mild: 0-5 - Moderate: 6-11 - Severe: 12-15 - Higher scores indicate greater symptom severity. OAB-q SS (Symptom Score): Range 0-25. - Mild: 0-8 - Moderate: 9-16 - Severe: 17-25 - Higher scores indicate worse symptoms. OAB-q HRQL (Health-Related Quality of Life): Range 0-36. - Mild: 0-12 - Moderate: 13-24 - Severe: 25-36 - Higher scores indicate worse quality of life.

Questionnaire	Item	Pre-vNOTES	Post-vNOTES	Post-Fotona Laser
OABSS	Daytime Frequency	2	2	0
	Nocturia	3	2	1
	Urgency	5	3	0
	Incontinence	5	3	0
	Total	15	10	1
OAB-q SS	Frequency (Daytime)	3	2	0
	Frequency (Nighttime)	3	2	1
	Urgency	4	3	0
	Incontinence (Urgency Incontinence)	4	3	0
	Other Incontinence	3	2	0
	Feeling of Incomplete Bladder Emptying	3	2	0
	Total Symptom Score	20	14	1
OAB-q HRQL	Fatigue	3	1	1
	Worry	3	1	0
	Impact on Social Activities	3	1	0
	Impact on Work and Daily Activities	3	1	0
	Impact on Enjoyment of Life	3	1	0
	Impact on Relationships	3	1	0
	Total Quality of Life Score	18	6	1

The OABSS, which assesses the severity of OAB symptoms such as daytime frequency, nocturia, urgency, and incontinence, showed a substantial reduction in scores from pre-vNOTES to post-vNOTES and further after Fotona laser treatment [12]. In the pre-vNOTES stage, the patient's OABSS score was 15, indicating severe OAB symptoms, characterized by frequent urination, significant nocturia, severe urgency, and incontinence. Following vNOTES, the OABSS score decreased to 10, reflecting a moderate improvement in symptoms such as urgency and incontinence. After Fotona laser treatment, the OABSS score dramatically reduced to 1, showing minimal symptoms, with the patient reporting no urgency or incontinence and only a mild instance of nocturia.

Similarly, the Overactive Bladder Questionnaire Symptom Score (OAB-q SS), which focuses on the frequency and severity of symptoms such as urgency and incontinence, declined from a total score of 20 in the pre-vNOTES period to 14 post-vNOTES [12]. This reduction suggests a noticeable improvement in symptoms, including a decreased frequency of both daytime and nighttime urination, and reduced urgency and incontinence. After Fotona laser treatment, the OAB-q SS score further decreased to 1, indicating a near-complete resolution of symptoms such as urgency and incontinence, along with a return to normal bladder emptying.

The Overactive Bladder Questionnaire Health-Related Quality of Life (OAB-q HRQL) evaluates how OAB symptoms affect patients’ quality of life in areas such as fatigue, worry, social activities, and relationships [12]. A pre-vNOTES score of 18 out of 36 showed a moderate impact on the patient's quality of life due to severe symptoms. This impact significantly reduced post-vNOTES, with a score of 6, indicating reduced worry and improved participation in daily and social activities. Following Fotona laser treatment, the OAB-q HRQL score further improved to 1, suggesting that the patient's quality of life was minimally affected, reflecting significant recovery. Overall, the comparison across pre-vNOTES, post-vNOTES, and post-Fotona laser treatment stages demonstrated a clear trajectory of improvement in both the severity of OAB symptoms and the quality of life of the patient. The data highlight the effectiveness of both vNOTES surgery and Fotona laser therapy in managing and significantly reducing OAB symptoms, with the Fotona laser showing a profound impact on achieving near-complete symptom resolution and enhancing daily well-being.

Cystoscopy

**Figure 8:**
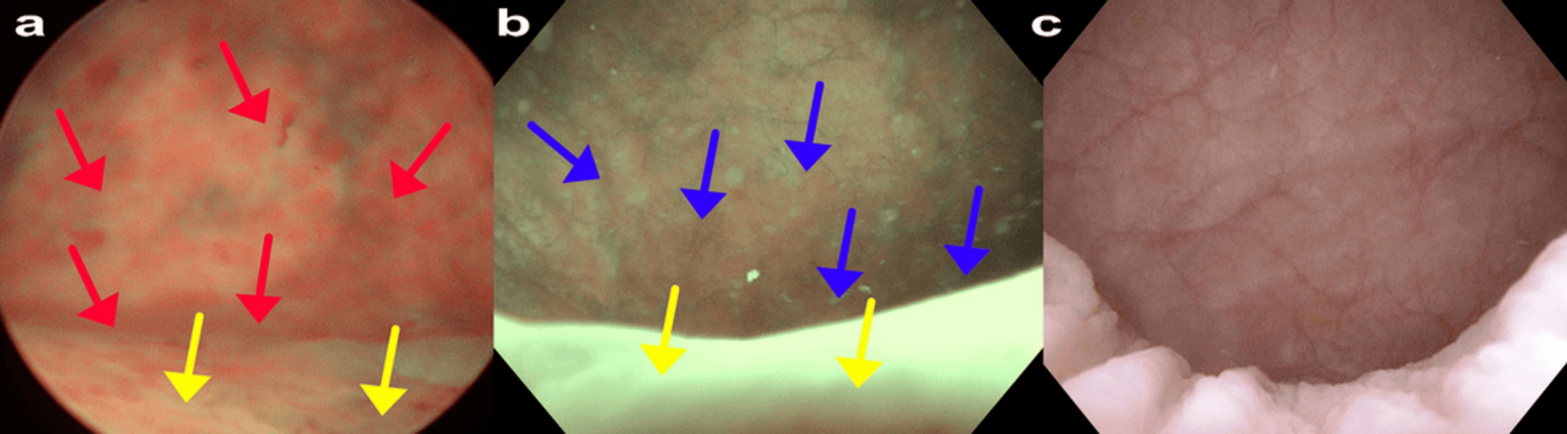
Cystoscopic Findings at Different Stages of Treatment a: Pre-vNOTES, b: Post-vNOTES, c: Post-Fotona laser treatment Red arrows: Inflammation throughout the bladder, prone to bleeding Yellow arrows: Edema Blue arrows: Improved condition, but some inflammation remains vNOTES: vaginal natural orifice transluminal endoscopic surgery

Figure 8 shows the cystoscopy findings at the different stages of treatment. In the pre-vNOTES condition (Figure 8a), significant inflammation was observed throughout the bladder, as indicated by red arrows. This inflammation was particularly pronounced in the areas adjacent to the polypropylene mesh. Additionally, severe edema was noted in the bladder, as indicated by yellow arrows. In the post-vNOTES condition (Figure 8b), a noticeable improvement in the overall bladder condition was observed. The previously severe inflammation was reduced, although some areas still exhibited inflammation, as indicated by the blue arrows. Edema is also present but is less severe compared to the pre-vNOTES stage, as indicated by the yellow arrows. This improvement suggests that the vNOTES procedure helped alleviate some irritation and inflammation caused by the mesh, although some residual effects remained. Post-Fotona laser treatment (Figure 8c) showed a significant improvement in bladder condition, with a substantial reduction in inflammation and edema. The bladder mucosa appeared normal with no visible signs of severe inflammation or edema. The findings indicate that the Fotona laser treatment has been highly effective in reducing the inflammatory response and improving the overall health of the bladder, achieving a near-complete resolution of the symptoms.

Vaginal bacterial culture and antibiotic susceptibility (reference data)

In this study, we conducted vaginal bacterial culture tests at each stage of treatment (pre-vNOTES, post-vNOTES, and post-Fotona Laser) and observed the types and quantities of bacteria detected. Table 2 shows the antibiotic susceptibility profiles of bacteria detected at each stage. These results were based on samples collected once at each stage and should be interpreted cautiously as reference data. They were evaluated at four levels: abundant, moderate, sparse, and not detected. In the pre-vNOTES test, *Corynebacterium sp.* was detected in abundant quantities, *Fusobacterium sp.* in moderate quantities, and the *Bacteroides fragilis* group in sparse quantities. The antibiotic susceptibility test results in Table 2 show that *Corynebacterium sp.* were resistant (R) to penicillin G (PCG), ampicillin (ABPC), clindamycin (CLDM), levofloxacin (LVFX), and flomoxef (FMOX). During the vNOTES procedure, cefmetazole (CMZ) was administered for five days and the foreign mesh material was removed. Post-vNOTES test results showed changes in the bacterial flora. *Corynebacterium sp. *continued to be detected in abundant quantities, and new sparse detection of anaerobic bacteria and *Gardnerella vaginalis* was observed. *Fusobacterium sp. *was not detected in the present study. The antibiotic susceptibility profiles in Table 2 show that *Corynebacterium sp.* remained resistant to PCG, CLDM, and LVFX. The post-Fotona laser treatment test revealed additional changes. All previously detected bacterial species were no longer detected, and *Lactobacillus spp. *appeared in moderate quantities. The antibiotic susceptibility profile in Table 2 shows increased sensitivity to PCG, ABPC, CMZ, and CLDM, and decreased resistance to LVFX and FMOX. The appearance of *Lactobacillus sp.* may indicate a healthy vaginal environment. This change might reflect the effect of Fotona laser treatment, but the influence of previously administered probiotics should also be considered.

**Table 2:** Antibiotic Susceptibility Profiles of Identified Bacteria at Different Treatment Phases S: Susceptible; I: Intermediate; R: Resistant PCG: Penicillin G; ABPC: Ampicillin; CVA/AMPC: Clavulanic acid/ampicillin; CTM: Cefotiam; CMZ: Cefmetazole; FMOX: Flomoxef; IPM/CS: Imipenem/cilastatin; EM: Erythromycin; CLDM: Clindamycin; MINO: Minocycline; VCM: Vancomycin; TFLX: Tosufloxacin; LVFX: Levofloxacin; CTX: Cefotaxime; vNOTES: vaginal natural orifice transluminal endoscopic surgery

Identified Bacteria	*Corynebacterium sp.* Pre-vNOTES	*Fusobacterium sp.* Pre-vNOTES	*Bacteroides fragilis* Group Pre-vNOTES	*Corynebacterium sp.* Post-vNOTES	Anaerobic Bacteria Post-vNOTES	*Gardnerella vaginalis* Post-vNOTES	*Lactobacillus sp.* Post- Fotona Laser
PCG	R	R	S	R	R	R	S
ABPC	R	S	S	R	R	S	S
CVA/AMPC	R	S	S	R	R	S	S
CTM	R	S	S	R	R	S	S
CMZ	S	S	S	S	S	S	S
FMOX	R	S	S	I	S	S	S
IPM/CS	R	S	S	I	R	R	S
EM	R	S	S	R	S	S	S
CLDM	R	S	S	R	R	S	S
MINO	S	S	S	S	S	S	S
VCM	S	S	S	S	S	S	S
TFLX	S	S	S	S	S	S	S
LVFX	R	S	S	I	S	S	S
CTX	S	S	S	S	S	S	S

## Discussion

This case report presents a novel management approach for specific complications following LSC, which is a common procedure for POP. The patient's MRI findings confirmed that the polypropylene mesh inserted during the LSC overlapped and formed a problematic mass. Comprehensive urodynamic testing revealed that the mesh mass stimulated involuntary bladder contractions when the bladder was urine-filled, causing significant discomfort and symptoms of OAB. To address this critical issue, the mesh mass was carefully removed using vNOTES, a minimally invasive technique. After mesh removal via vNOTES, the patient's symptoms noticeably improved, but significant tissue damage remained due to the extensive mesh removal process. To address this residual tissue damage and promote healing, Fotona laser therapy, a cutting-edge treatment modality, was used. This innovative combined approach of mesh removal by vNOTES followed by Fotona laser therapy ultimately proved highly effective in completely resolving the patient's persistent urinary symptoms and improving the overall quality of life.

LSC using polypropylene mesh has become a standard treatment for POP [1]; however, risks of complications, such as those in this case, exist and can significantly impact patient quality of life. These symptoms include chronic pelvic pain, mesh-related infections, OAB, and persistent vaginitis [2,3]. Long-term studies have shown that complications after LSC can persist for years, potentially requiring ongoing medical intervention, with reoperation rates reaching 5.1% over four years for various reasons, including mesh-related issues and recurrent prolapse [6]. These findings emphasize the critical need for effective management of post-LSC complications. The innovative approach used in this case demonstrates a promising possibility of improving patient outcomes.

The vNOTES approach offers several advantages over conventional methods for mesh removal, particularly improved visibility and access to mesh fixation sites, while minimizing surgical trauma [6,7]. The success of mesh removal via vNOTES led to significant improvements in patients' OABSS and OAB-q scores, demonstrating the effectiveness of this minimally invasive technique in managing post-LSC complications.

However, a noteworthy observation in this case was the changes in vaginal bacterial flora across the different treatment phases. It's important to preface this by stating that these results were based on single cultures taken at each stage (pre-vNOTES, post-vNOTES, and post-Fotona laser), and should therefore be interpreted cautiously as reference data rather than definitive findings. With this caveat in mind, the bacterial cultures showed interesting trends. Despite the initial improvement in OAB symptoms after vNOTES, the patient continued to experience persistent vaginitis that was unresponsive to conventional treatments. Pre-laser therapy culture revealed a mixed infection, including antibiotic-resistant strains of *Corynebacterium sp.*, *Fusobacterium sp.*, and *B. fragilis*. This complex microbial environment has been recognized as a significant challenge in recent bacteriological treatments [13,14]. Interestingly, the follow-up culture at one-month post-Fotona laser treatment showed a shift towards normalization of vaginal flora, with predominant growth of *Lactobacillus spp.* and absence of previously detected anaerobic bacteria. While this shift from a pathogenic, antibiotic-resistant microbial community to a *Lactobacillus*-dominated flora is intriguing in the context of post-LSC management, it's important to note that this observation is based on limited data and requires further investigation. Studies have consistently demonstrated that healthy vaginal flora is associated with the presence of *Lactobacillus spp.* [15-17]. However, the significance of these microbiota changes in the context of post-LSC management requires more comprehensive research with larger sample sizes and longitudinal monitoring to draw definitive conclusions.

Comprehensive assessment of the patient's condition using various diagnostic methods, such as MRI, urodynamic testing, and cystoscopy, provided valuable insights into treatment progress. MRI findings after vNOTES showed improvement in the cystocele and surrounding tissues, whereas urodynamic testing after Fotona laser treatment demonstrated significant improvements in bladder function and control. The cystoscopy results also confirmed a reduction in bladder inflammation and edema, supporting the effectiveness of this combined treatment approach.

The dramatic improvement in the patient's quality of life, as reflected in the OAB-q HRQL scores, demonstrates the potential of this approach in restoring the patient's health. The near-complete resolution of OAB symptoms after Fotona laser treatment was consistent with the results of previous studies [12].

Several studies have reported promising results regarding the effects of Fotona laser treatment on genitourinary symptoms. A combination treatment of Er:YAG and Nd:YAG lasers for urinary incontinence in elite female athletes showed significant improvement in symptoms, with effects lasting for two years [18]. Similar combination therapy has also been reported to be effective for interstitial cystitis/bladder pain syndrome and vulvodynia, with pain scores decreasing from 8 to 2 and urination frequency reducing from 20 to 8 times per day after treatment [19]. Furthermore, combined therapy with non-ablative Er:YAG and Nd:YAG lasers has shown effectiveness in improving anal incontinence and vaginal atrophy, with reported increases in Vaginal Health Index scores and improvements in anal incontinence severity index [20].

## Conclusions

This case report demonstrates the potential of a new management strategy for specific complications following LSC, particularly OAB symptoms caused by abnormal mesh placement or tissue effects. The combination of mesh removal via vNOTES and Fotona laser therapy may offer a minimally invasive solution that addresses both the mechanical and microbiological aspects of post-LSC complications.
